# Endometrial Tumor Classification by Histomorphology and Biomarkers in the Nurses' Health Study

**DOI:** 10.1155/2021/8884364

**Published:** 2021-03-12

**Authors:** Jaclyn C. Watkins, Michael J. Downing, Marta Crous-Bou, Evan L. Busch, Maxine Chen, Immaculata De Vivo, George L. Mutter

**Affiliations:** ^1^Department of Pathology, Brigham and Women's Hospital, 75 Francis Street, Boston, MA, USA 02115; ^2^Harvard Medical School, 25 Shattuck St., Boston, MA, USA 02115; ^3^Channing Division of Network Medicine, Brigham and Women's Hospital, 181 Longwood Avenue, Boston, MA, USA 02115; ^4^Department of Epidemiology, Harvard T.H. Chan School of Public Health, 677 Huntington Ave., Boston, MA, USA 02115

## Abstract

**Objective:**

Endometrial cancers have historically been classified by histomorphologic appearance, which is subject to interobserver disagreement. As molecular and biomarker testing has become increasingly available, the prognostic significance and accuracy of histomorphologic diagnoses have been questioned. To address these issues for a large, prospective cohort study, we provide the results of a centralized pathology review and biomarker analysis of all incidental endometrial carcinomas occurring between 1976 and 2012 in the Nurses' Health Study.

**Methods:**

Routine histology of all (*n* = 360) cases was reviewed for histomorphologic diagnosis. Cases were subsequently planted in a tissue microarray to explore expression of a variety of biomarkers (e.g., ER, PR, p53, PTEN, PAX2, AMACR, HNF1*β*, Napsin A, p16, PAX8, and GATA3).

**Results:**

Histologic subtypes included endometrioid (87.2%), serous (5.6%), carcinosarcoma (3.9%), clear cell (1.7%), and mixed type (1.7%). Biomarker results within histologic subtypes were consistent with existing literature: abnormal p53 was frequent in serous cases (74%), and HNF1*β* (67%), Napsin A (67%), and AMACR (83%) expression was frequent in clear cell carcinomas. Our dataset also allowed for examination of biomarker expression across non-preselected histologies. The results demonstrated that (1) HNF1*β* was not specific for clear cell carcinoma, (2) *TP53* mutations occurred across many histologies, and (3) GATA3 was expressed across multiple histotypes, with 75% of positive cases demonstrating high-grade features.

**Conclusions:**

Our findings establish the subtypes of endometrial cancer occurring in the Nurses' Health Study, corroborate the sensitivity of certain well-established biomarkers, and call into question previously identified associations between certain biomarkers (e.g., HNF1B) and particular histotypes.

## 1. Introduction

Endometrial cancers have historically been subclassified by their histomorphologic appearance alone [1, 2]. However, histomorphologic classification suffers from interobserver agreement issues, particularly in the case of high-grade endometrial carcinomas (e.g., FIGO grade 3 endometrioid, clear cell carcinoma, serous carcinoma, and carcinosarcoma) [3–7]. Further, in the molecular era, morphologic subtyping is considered insufficient for determining prognosis. Most famously, The Cancer Genome Atlas molecularly classified endometrial tumors into four categories which were prognostically significant irrespective of histomorphology [8].

Despite the inherent limitations in morphologic analysis of tumors, histomorphologic diagnosis largely determines inclusion in cancer registries as well as in clinical and epidemiologic research [9]. These diagnoses may suffer from changes in diagnostic categories over time, an absence of centralized pathology review, and, importantly, the absence of corroborative biomarker (immunohistochemical) data.

The application of biomarkers (i.e., immunohistochemical staining) assists pathologists in rendering more accurate diagnoses. A recent study found that up to 40% of cases initially diagnosed as high-grade endometrial endometrioid adenocarcinomas may be reclassified as uterine serous carcinomas based on a panel of four immunostains (p53, p16, estrogen receptor (ER), and mammaglobin) [10]. Immunostains in routine practice, however, are often not pursued due to a pathologist's degree of morphologic certainty or the possibility of ambiguous results.

Further, biomarkers have their own limitations. Biomarker sensitivity and specificity have largely been determined by research conducted on highly selected cohorts of tumors, typically including only those with unambiguous morphologies. Thus, the sensitivity and specificity of markers tends to shift once the markers are applied in the clinical setting and to cases with less prototypical morphologies. For example, hepatocyte nuclear factor-1beta (HNF1*β*) expression was initially thought to have a specificity of 93 to 100% for clear cell carcinoma [11–14]; however, overtime, the specificity has been questioned as expression has been documented in a subset of serous and endometrioid carcinomas [15–17].

In this study, we attempt to address two of the aforementioned issues. First, we perform a centralized review of the incidental endometrial cancer cases arising in the Nurses' Health Study, a large, prospective cohort study with long-term follow-up. Secondly, we attempt to systematically apply a set of biomarkers to incidental endometrial cancer cases, thus exploring expression across a spectrum of subtypes. We explore biomarkers that are currently well-established in their interpretation (e.g., p53) as well as more novel biomarkers (e.g., GATA3). Further, we explore the limitations and benefits of applying biomarkers retrospectively in the case of a large epidemiologic study, especially the feasibility of interpretation in the setting of tissue microarrays.

## 2. Materials and Methods

This study was approved by the Human Studies Review Board at Brigham and Women's Hospital. The protocol for this study was approved by the Human Research Committees at Brigham and Women's Hospital, Boston, MA, USA.

### 2.1. Case Selection

Using data from the Nurses' Health Study [18, 19], a prospective cohort of 121,700 female nurses initiated in 1976, all incidental endometrial cancers diagnosed between 1976 and 2012 were identified.

When available, paraffin-embedded tissue and hematoxylin and eosin stained slides from the patient's endometrial biopsy or hysterectomy were obtained. Only cases with paraffin blocks for the creation of tissue microarrays were included. Full hematoxylin and eosin sections of the original clinical diagnostic blocks underwent central pathology review by a pathologist (GM), and a histologic diagnosis was rendered for each case using current diagnostic criteria [1]. Additional information regarding case ascertainment and tissue microarray construction has been published elsewhere [20].

For each specimen, a representative paraffin-embedded tissue block was chosen, and three 0.6 mm cores were planted in a tissue microarray. Serial sections of each microarray were used for marker studies as below.

### 2.2. Immunohistochemical Studies

For each marker studied, four-micron thick sections of each tissue microarray were stained in the following sequence: hematoxylin and eosin, marker replicate 1, pankeratin AE1/AE3 (Dako Cat#M3515 at 1 : 200 dilution), and marker replicate 2. The following immunostains were performed using the Leica Bond III staining platform using the following primary antibodies: estrogen receptor (ER, murine monoclonal ERID5 (Dako) at 1 : 300 dilution), progesterone receptor (PR, murine monoclonal PgR636 (Dako) at 1 : 150 dilution), p53 (murine monoclonal PAb 1801 (Leica Biosystems) at 1 : 300 dilution), AMACR (rabbit monoclonal Z2001 (Zeta Corp) at 1 : 50), ARID1A (rabbit polyclonal HPA005456 (Sigma) at 1 : 200 dilution), GATA3 (murine monoclonal L50-823 (Biocare) at 1 : 100 dilution), HNF1*β* (rabbit polyclonal HPA002083 (Sigma) at 1 : 500 dilution), Napsin A (murine monoclonal NCL-L-Napsin A (Leica) at 1 : 400 dilution), p16 (murine monoclonal E6H4 (Ventana) at 1 : 300 dilution), PAX2 (polyclonal rabbit Z-RX2 (InVitrogen) at 1 : 300 dilution), PAX8 (rabbit polyclonal 10336-1-AP (Proteintech) at 1 : 600 dilution), and PTEN (murine monoclonal 6h2.1 (Millipore) at 1 : 100).

### 2.3. Marker Interpretation

All stained slides were digitally captured at 40x magnification by a Hamamatsu nanozoomer whole slide digital scanner. For each marker, hematoxylin and eosin and keratin-stained images of matched tissue microarray sections were used to confirm the presence of tumor tissue and to discriminate tumor cells from background as needed. Duplicate stains for each marker were independently scored for marker specific signal within tumor cells (JW and GM) (see Table 1 for scoring methodology). Discordant replicates were resolved by rereview. Markers scored on continuous scales were averaged across the duplicate runs for final data analysis. Technical failures due to tissue dropout, high background, or low signal were excluded as noninformative on a marker-by-marker basis.

Due to the limited tumor represented on the tissue microarrays, p53 was only interpreted as abnormal when staining was diffuse and strong; a “null” phenotype was not reported due to the possibility of failed staining or regional variability in tumor expression and thus the possibility of false positives [21].

### 2.4. Case Inclusion and Exclusion Criteria

Some patients had separate endometrial biopsies and hysterectomy specimens containing tumor. After staining, only unique specimens (1 per patient) were included in the final analysis, with preference for hysterectomy specimens over biopsies. Nonepithelial tumors were excluded. Carcinosarcoma and mixed carcinoma cases were included.

### 2.5. Covariates

Age at diagnosis (continuous) and body mass index at diagnosis [22] (continuous, kg/m^2^) were assessed from the last biennial Nurses' Health Study questionnaire prior to cancer diagnosis.

### 2.6. Statistical Analyses

Statistical analysis was performed in SYSTAT (v13.1, Systat Software, Inc., San Jose, CA). One-way ANOVA was used to independently compare age and body mass index with the diagnostic tumor subtypes. *t*-tests, with separate variances, were employed to compare biomarker expression across age and body mass index. Results with a *p* value < 0.05 were considered statistically significant. Hierarchical clustering of bimodally scored (normal vs. abnormal) marker results was performed using Ward's linkages and Jaccard similarity coefficient distance metric. Bimodal scoring of the continuous percentages of estrogen and progesterone receptor was done across a 10% threshold.

## 3. Results

Pathology materials were received for 472 patients with reported endometrial cancer. After application of the inclusion criteria, 360 patients had adequate tissue for inclusion in the tissue microarray (343 hysterectomy specimens and 17 biopsy/curettages).

### 3.1. Histomorphologic Review

Histomorphologic diagnoses were as follows: 314 endometrioid (87.2%), 20 serous (5.6%), 14 carcinosarcoma (3.9%), 6 clear cell (1.7%), and 6 mixed endometrioid plus nonendometrioid type (1.7%). Within the endometrioid group, 244 were grade 1, 43 were grade 2, and 27 were grade 3.

### 3.2. Biomarker Results within Histomorphologic Diagnoses (Table 2)

Clear cell carcinomas frequently expressed AMACR, HNF1B, and Napsin A (83.3%, 66.7%, and 66.7%, respectively). Serous carcinomas commonly displayed diffuse, strong p53 staining (mutant phenotype; 73.7%) and p16 diffuse positivity (70%). Endometrioid tumors demonstrated high rates of ER (92.7%) and PR (85.1%) expression, with the highest ER and PR expression noted in FIGO grade 1 endometrioid tumors.

### 3.3. Biomarker Expression across Histomorphologic Diagnoses

**Figure 1:**
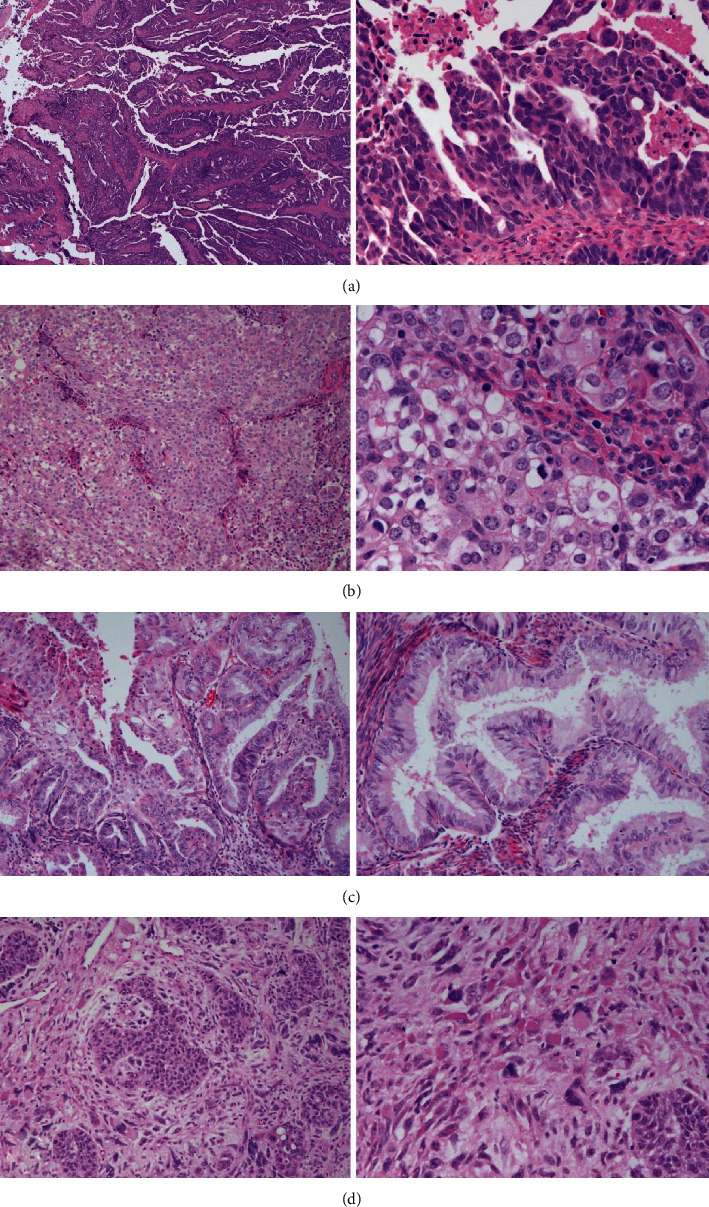
Four examples ((a–d), each a separate case) of GATA3-positive tumors with nonmesonephric histology. (a) Serous. (b) Ambiguous morphology, most consistent with grade 3 endometrioid carcinoma. (c) Conventional grade 1 endometrioid. (d) Carcinosarcoma.

GATA3 was expressed across histotypes (16 cases total; 10 endometrioid (62.5%), 3 serous (18.8%), 2 carcinosarcoma (12.5%), and 1 clear cell (6.25%)). GATA3 expression correlated positively with expression of HNF1*β* (*p* = 0.009) and p16 (*p* = 0.017) and absence of staining for PAX2 (*p* = 0.002), ER (*p* < 0.001), and PR (*p* < 0.001). Rereview of the GATA3 positive tumors revealed an absence of morphologic findings consistent with mesonephric carcinomas. Further, 12 of the 16 cases showed high-grade morphology with ambiguous cytology (Figure 1). GATA3 and PAX8, respectively, markers of mesonephric and Müllerian origin, were not always mutually exclusive. 79.6% of cases (262/329) were positive for only one of the two markers, 15.8% (52/329) were negative for both, and 4.6% of cases (15/329) were positive for both.

Diffuse, strong p53 expression (mutant phenotype) was seen across histotypes. Of p53 mutant tumors (37 total), 37.8% were serous, 32.4% were endometrioid, 13.5% were carcinosarcoma, 8.1% were clear cell carcinoma, and 8.1% were mixed type. HNF1*β* showed similar lack of correlation with a specific histologic type with 71.9% of positive cases being endometrioid, 10.5% serous, 7.0% carcinosarcoma, 7.0% clear cell carcinoma, and 3.5% mixed type. Stated another way, 33.3% of carcinosarcomas, 66.7% of clear cell carcinomas, 13.7% of endometrioid carcinomas, 40% of mixed type tumors, and 31.6% of serous carcinomas stained positively for HNF1*β*.

Mutant p53 staining had variable correlation with aberrant p16 staining, a previously reported association especially in serous cancers [23]. In our overall cohort, p53 and p16 stainings were discordant in 8.9% of cases (31/347). Of these cases, 48.3% (15/31) demonstrated mutant p53 with only patchy p16 staining.

### 3.4. Marker Expression Patterns

**Figure 2:**
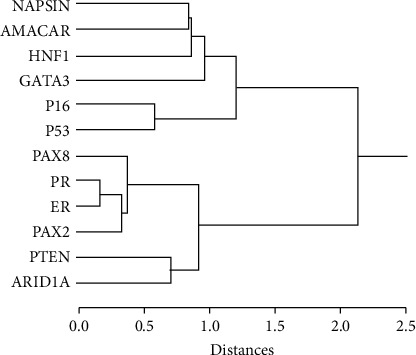
Self-organized hierarchical clustering of 12 biomarkers in 274 endometrial cancers. Dendrogram shows degree of marker association (distance measure) in 274 cases with complete data for all markers (cases with partial missing data excluded for computational reasons). Estrogen (ER) and progesterone (PR) receptor results scored dichotomously across a 10% threshold. Two major clusters are evident, containing P53 and PTEN, respectively. Ward's linkage method and Jaccard similarity coefficient distance metric for binary data. Distal branches, such as those containing ER + PR and p16 + p53, indicate a level of association between members of the limb.

Hierarchical clustering of biomarker results from 240 cases with complete data shows two main clades corresponding to PTEN and P53 mutant classes (Figure 2).

### 3.5. Age, Body Mass Index, and Biomarker Expression

Age at diagnosis was found to be statistically different across histologic categories (*p* = 0.02, Table 3), with endometrioid type tumors presenting on average at a younger age (mean = 68.5 y) than other subtypes. Body mass index did not differ significantly across diagnostic categories (*p* = 0.1, Table 3).

When body mass index and age were compared with biomarker expression (Table 4), several significant associations were found. Body mass index was significantly lower in women whose tumors expressed HNF1*β* (26.9 vs. 30.5 kg/m^2^, *p* < 0.001), Napsin A (25.1 vs. 30.0 kg/m^2^, *p* = 0.007), and mutant p53 (26.0 vs. 30.3 kg/m^2^, *p* < 0.001). Body mass index was significantly higher in women whose tumors expressed ER (30.3 vs. 27.2 kg/m^2^, *p* = 0.001) and PR (30.4 vs. 28.2 kg/m^2^, *p* = 0.033). The loss of expression of PAX2 was associated with younger age at diagnosis (68.6 vs. 70.5, *p* = 0.035).

## 4. Discussion

Our study addresses two key issues. First, we performed a centralized review of the incidental endometrial cancers in the Nurses' Health Study. Secondly, through the administration of biomarkers to a large, incidental cohort, we explored biomarker expression across a spectrum of endometrial tumors with variable morphologies.

As was expected, most incidental cases of endometrial cancer in the Nurses' Health Study (87.2% of cases) were “type 1 endometrial carcinomas” (i.e., endometrioid adenocarcinomas arising secondary to estrogen stimulation). Such “type 1” cancers account for the majority of endometrial tumors and typically arise in obese, post-menopausal women [24–26]. “Type II endometrial carcinomas” (i.e., predominantly nonendometrioid carcinomas arising in the setting of *TP53* mutation or 1p deletion) [25–27], were less common in our cohort, reflecting their lower prevalence in the general population.

Our biomarker exploration of these tumors confirmed the preexisting associations between type I and type II endometrial carcinomas with hormonal receptors and p53 expression. Low-grade endometrioid carcinomas (type I) expressed high rates of ER and PR, supporting the hormonal pathway to carcinogenesis. Likewise, our type 2 tumors (nonendometrioid) demonstrated frequent p53 overexpression (mutant phenotype). Such findings are not novel, but do support the feasibility and reliability of applying biomarkers to large epidemiologic studies in a retrospective fashion.

While we confirmed preexisting biomarker expression pattern within histotypes, the advantage of our study was the ability to assess biomarkers across an incidental cohort of histomorphologic subtypes. Multiple marker-histotype correlations were found to be less specific than previously thought. Specifically, while p53 abnormalities are seen in 73.7% of serous cancers, 62.2% of all p53 abnormal cancers had a nonserous histology. This experience is mirrored in other purely marker-driven endometrial cancer classification systems such as the genomic-based TCGA classification schema, where molecular and histopathologic classes only partly overlap, and some unique molecular phenotypes of clinical interest (such as polymerase E mutation) cannot be reliably identified by histology alone [8, 16].

Our study additionally contributes to the growing evidence that HNF1*β* is a nonspecific marker [15–17]. While two-thirds of our clear-cell cases stained with HNF1*β*, 31.6% of serous carcinomas and 13.7% of endometrioid carcinomas expressed HNF1*β*, making HNF1*β* staining an unreliable marker in the clear cell versus serous/endometrioid differential. Thus, we conclude that HNF1*β*, on its own, should not be considered a specific marker for clear cell carcinoma in clinical practice.

Our findings regarding expression of GATA3 are also of interest. Prior studies demonstrate GATA3 be a highly sensitive and specific marker for mesonephric lineage in lesions of the lower female genital tract [28, 29]. In our study, GATA3 positive staining in an unselected sample of uterine tumors did not correspond well to any single histotype. Notably, none of the cases, even those of low-grade cytology, displayed the classic morphologic features of mesonephric carcinomas (e.g., small round tubules, cuboidal or flatted epithelium, and angulated vesicular nuclei). Furthermore, 12 of 16 positive cases displayed high-grade or ambiguous cytologic features, raising the possibility that poorly differentiated endometrial tumors may express GATA3 not due to lineage but rather due to gain of function mutations. This is further demonstrated by our finding that 4.6% of cases demonstrated positivity for both PAX8 and GATA3, two stains that should be mutually exclusive of each other.

An unsupervised self-organizing dendrogram of marker results in our tumors gives an indication of how combinatorial marker trends can define tumor subgroups (Figure 2). Two major clusters are evident: a p53 mutant arm and a PTEN/PAX2 arm, corresponding to “type II” and “type I” endometrial cancers, respectively. Further, there is a p16/p53 subgroup distinct from a Napsin A/AMACR/HNF1*β* class. Of note, GATA3 expression clustered with both clear cell markers and/or p53/p16 expression. This clustering provides additional evidence that GATA3 expression likely denotes aberrant expression/gain of function mutations in high-grade malignancies arising from mutations in *TP53* rather than mesonephric lineage.

Given our unique dataset, we were able to compare biomarker expression across participants' ages and body mass index. As expected, p53 expression was associated with significantly lower body mass index, and ER/PR expression was associated with significantly higher body mass index (additional ER/PR findings in this cohort are reported elsewhere [20]). Additionally, endometrioid-type tumors tended to present at earlier ages than other subtypes. These findings are consistent with common mechanisms of endometrial carcinogenesis in which either hormonal stimulation (typically in the setting of obesity) [30] or p53 mutation leads to neoplastic growth [25, 26, 31, 32].

Our study did highlight some of the necessities and limitations of working with tissue microarrays. Of note, when performing a centralized pathology review, we found it essential to render a morphologic diagnosis on whole H&E slides only, not on the tissue microarray preparations. This is due to the limited amount of tumor present on the tissue microarray. Secondly, due to the occasional absence of internal controls noted in the tissue microarray tissues, we recommend running separate positive controls on each tissue microarray assay. We also recommend replicate stain reads to control for idiosyncratic runs, ideally with the replicates reviewed independently by mutually blinded pathologists. The main limitation of using tissue microarrays in our study was the inability to reliably interpret p53 protein null phenotypes caused by rare nonsense mutations [33]. Thus, more of our cases likely had aberrant p53 staining than we could reliably conclude from our tissue microarray preparations.

In this study, we provide a centralized review of the incidental endometrial cancers in the Nurses' Health Study as well as the biomarker expression results across this cohort. This data is largely applicable to future clinical/epidemiologic studies that derive data on endometrial cancers form the Nurses' Health Study. However, we were also able to test the hypothesis that biomarker studies in which cases are preselected by pathologists likely lead to overestimated sensitivities and specificities. In the case of GATA3, p53, and HNF1*β*, we found this hypothesis to be true. We believe our approach of testing biomarkers across an incidental population of cancers gives a more realistic sense of the utility of such markers when applied to incidental disease. Further, our findings mimic those of The Cancer Genome Atlas and other data collected from next-generation sequencing in which there is much overlap in the mutational profiles of tumor categories as defined by histomorphology. We conclude that expression of a single biomarker (e.g., HNF1*β* and GATA3) should not be interpreted as diagnostic of a particular tumor type without taking into consideration the histology. We would recommend that, when in doubt of the histology, a panel of immunostains, including stains redundant for a diagnosis (e.g., PTEN, ARID1a, and ER for endometrioid adenocarcinoma; HNF1*β*, Napsin A, and AMACR for clear cell carcinoma; and p16 and p53 for serous carcinoma), be performed rather than relying on a single immunostain. Lastly, we show that tissue microarrays have some limitations; however, the results of biomarker studies in this setting are largely reliable and reproducible.

## Figures and Tables

**Table 1:** Biomarker scoring and rationale for inclusion.

Marker	Rationale	#scoring
AMACAR	Candidate clear cell type marker	0 = WT (neg), 1 = pos, 3 = NI
ARID1A	Candidate clear cell type marker	0 = WT (pos), 1 = lost (neg), 3 = NI
Hnf1beta	Candidate clear cell type marker	0 = WT (neg), 1 = pos, 3 = NI
Napsin A	Candidate clear cell type marker	0 = WT (neg), 1 = pos, 3 = NI
p16	Candidate serous type marker	0 = WT (neg or patchy), 1 = diffusely pos, 3 = NI
ER	Hormone responsiveness	Percentage positive nuclear staining: 0 = neg (<10% staining), 1 = pos (≥10% staining); also
PR	Hormone responsiveness	Percentage positive nuclear staining: 0 = neg (<10% staining), 1 = pos (≥10% staining)
PAX8	Müllerian (paramesonephric) lineage marker	0 = neg, 1 = pos, 3 = NI
PAX2	Primary inactivation mechanism	0 = WT (pos), 1 = lost (neg), 3 = NI
p53	Primary mutation mechanism	0 = WT, 1 = diffuse, strong (mutated) expression 3 = NI
PTEN	Primary mutation mechanism	0 = WT (pos), 1 = lost (neg), 3 = NI
GATA3	Wolffian (mesonephric) lineage marker	0 = WT (neg), 1 = pos, 3 = NI

^#^WT = wild type; NI = not informative; pos = positive; neg = negative.

**Table:** (a) Reportable marker results in 360 tumors studied

	Immunomarker, scored results											
	AMACR	ARID1A	HNF1B	ER	GATA3	Napsin A	P16	P53	PAX2	PAX8	PR	PTEN
	POS	NEG	POS	>10%	POS	POS	POS	Mutant	NEG	POS	>10%	NEG
Total cases stained	360	360	360	360	360	360	360	360	360	360	360	360
Total excluded	19	23	19	12	20	16	10	4	13	24	16	52
Total # informative	341	337	341	348	340	344	350	356	347	336	344	308
Total # abnormal	48	115	57	300	16	6	37	37	253	282	264	124
% abnormal	14.1	34.1	16.7	86.2	4.7	1.7	10.6	10.4	72.9	83.9	76.7	40.3

**Table:** (b) Distribution of reportable marker results, by tumor histotype

Histotype	Immunomarker scored results, informative cases % (*n*/total)											
	AMACR	ARID1A	HNF1*β*	ER	GATA3	Napsin A	P16	P53	PAX2	PAX8	PR	PTEN
Endometrioid	13.4% (40/299)	37.2% (110/296)	13.7% (41/299)	92.7% (281/303)	3.3% (10/299)	0% (0/302)	4.2% (13/306)	3.9% (12/311)	75.5% (228/302)	82.7% (243/294)	85.1% (256/301)	43.4% (116/267)
Serous	5.3% (1/19)	5.6% (1/18)	31.6% (6/19)	55% (11/20)	15.8% (3/19)	0% (0/19)	70% (14/20)	73.7% (14/19)	55% (11/20)	95% (19/20)	26.3% (5/19)	11.8% (2/17)
Carcinosarcoma	8.3% (1/12)	16.7% (2/12)	33.3% (4/12)	30.8% (4/13)	16.7% (2/12)	8.3% (1/12)	66.7% (8/12)	35.7% (5/14)	76.9% (10/13)	81.8% (9/11)	15.4% (2/13)	25% (3/12)
Clear cell carcinoma	83.3% (5/6)	0% (0/6)	66.7% (4/6)	33.3% (2/6)	16.7% (1/6)	66.7% (4/6)	0% (0/6)	50% (3/6)	33.3% (2/6)	100% (6/6)	16.7% (1/6)	16.7% (1/6)
Mixed type	20% (1/5)	40% (2/5)	40% (2/5)	33.3% (2/6)	0% (0/4)	20% (1/5)	33.3% (2/6)	50% (3/6)	33.3% (2/6)	100% (5/5)	0% (0/5)	33.3% (2/6)

^∗^Percentages are calculated for each marker (columns) by histotype (rows). Scorable results only, noninformative cases excluded.

**Table 3:** Mean age and body mass index at presentation by tumor histotype.

Tumor histotype	Mean age (years)	Body mass index (kg/m^2^)
Endometrioid	68.5	30.3
Serous	72.7	27.5
Carcinosarcoma	73.3	26.8
Clear cell carcinoma	71.4	25.2
Mixed type	69.8	28.6
*p* (Kruskal-Wallace)	0.11	0.115

**Table 4:** Mean Age and Body Mass Index at Presentation by Marker Status (p, t-test separate variance).

	Age	Age	Age	Body mass index	Body mass index	Body mass index	Comment
Marker	Wild type	Mutant	*p*	Wild type	Mutant	*p*	
AMACR	69.0	68.7	0.775	29.8	30.0	0.923	
ARID1A	69.2	68.6	0.539	30.3	29.2	0.160	
HNF1*β*	68.8	69.9	0.354	30.5	26.9	<0.001	
Napsin A	68.9	73.0	0.357	29.9	25.1	0.007	
p16	68.9	70.5	0.178	30.0	29.5	0.771	
ER	70.7	68.8	0.111	27.2	30.3	0.001	>10% is wild type
PR	69.6	68.8	0.402	28.3	30.4	0.033	>10% is wild type
PAX8	69.0	69.0	1.000	31.6	29.5	0.067	
PAX2	70.5	68.6	0.035	29.1	30.1	0.252	
p53	68.9	70.3	0.336	30.3	26.0	<0.001	
PTEN	69.9	68.4	0.077	29.8	29.9	0.979	
GATA3	68.9	71.1	0.290	30.0	26.9	0.108	

## Data Availability

The Nurses' Health Study data are housed within the Harvard School of Public Health and are not publicly available without an IRB and application for access.

## Additional Points

*Highlights*. (1) This study characterizes all incidental endometrial cancers in the Nurses' Health Study. (2) Our findings call into question specificity of several commonly used biomarkers. (3) We demonstrate that tissue microarrays are reliable and reproducible in retrospective studies.
